# Survival and Closure by Secondary Intention of a Pharyngocutaneous Fistula After Fulminant Descending Necrotizing Mediastinitis

**DOI:** 10.7759/cureus.107585

**Published:** 2026-04-23

**Authors:** Vanessa J Zhang, Mark W Maxfield, Christopher J Ito

**Affiliations:** 1 Otolaryngology, UMass Chan Medical School, Worcester, USA; 2 Surgery, UMass Chan Medical School, Worcester, USA

**Keywords:** actinomycosis, botulinum toxin, conservative management, deep neck infection, descending necrotizing mediastinitis, pharyngocutaneous fistula

## Abstract

Descending necrotizing mediastinitis is a rare, life-threatening extension of deep neck infection. We report the case of a 51-year-old woman who initially presented with a sore throat and was treated as uncomplicated pharyngitis before rapidly developing neck swelling, chest pain, dyspnea, septic shock, pneumomediastinum, and bilateral pleural empyemas. Combined cervicothoracic exploration showed dishwater fluid in the neck and mediastinum without esophageal perforation, and cultures grew *Streptococcus anginosus* and mixed oral flora with *Actinomyces odontolyticus* bacteremia. Her course was complicated by stress-induced cardiomyopathy, acute kidney injury requiring continuous renal replacement therapy, and vasopressor-associated digital ischemia. On hospital day 17, repeat exploration identified a large left hypopharyngeal pharyngocutaneous fistula communicating with the open neck wound. Because of critical illness and poor peripheral perfusion, flap reconstruction was deferred. The fistula was managed with repeated antiseptic packing, strict gastrostomy feeding, salivary suctioning, glycopyrrolate, scopolamine, and later botulinum toxin injections to the parotid and submandibular glands. The defect gradually contracted and closed by secondary intention over approximately 10 weeks. This case highlights that, in carefully selected high-risk patients, structured conservative management with salivary suppression may allow closure of a large pharyngocutaneous fistula after fulminant descending necrotizing mediastinitis.

## Introduction

Descending necrotizing mediastinitis (DNM) is an uncommon but life-threatening extension of deep neck infection in which bacteria spread along cervical fascial planes into the mediastinum [1-4]. Despite modern imaging, antibiotics, and intensive care, reported mortality remains in the double digits [2-7]. Early CT imaging of the neck and chest, rapid initiation of broad-spectrum antibiotics, and combined cervical and mediastinal drainage are consistently associated with improved outcomes [1-4,6,7].

Pharyngocutaneous fistula is most familiar after total laryngectomy, particularly in patients with prior radiotherapy, malnutrition, anemia, or wound infection [8-11]. Large or persistent fistulas in infected or irradiated fields have traditionally been managed with regional or free tissue transfer [8,11], but non-flap strategies, including meticulous local wound care, enteral diversion, salivary suppression, and adjuncts such as negative pressure therapy, can achieve closure in selected patients [8-13]. Evidence is limited for pharyngocutaneous fistula arising in the setting of DNM [14-18]. We report a patient with fulminant DNM complicated by a large hypopharyngeal pharyngocutaneous fistula that closed by secondary intention with a structured conservative strategy supplemented by salivary chemodenervation.

## Case presentation

A 51-year-old woman with a history of papillary thyroid carcinoma treated eight years earlier with total thyroidectomy and radioactive iodine initially presented to urgent care after one day of left-sided sore throat. At that visit, she reported odynophagia but no fever, cough, congestion, rhinorrhea, ear pain, abdominal pain, or fatigue. Examination showed posterior oropharyngeal erythema without obvious neck swelling or airway distress, and rapid streptococcal testing was negative. She was treated conservatively for presumed uncomplicated pharyngitis.

The following day, she presented to an emergency department with a persistent sore throat, painful swallowing, anterior neck discomfort, and a sensation that saliva was “stuck” in the back of her throat. She also described dyspnea and pain extending into the upper chest. Examination was notable for tenderness and lymph node swelling in the anterior cervical chain, greater on the left, but there was no drooling, trismus, stridor, limitation of neck motion, or other clear evidence of peritonsillar abscess, retropharyngeal abscess, or impending airway compromise. She improved transiently after dexamethasone and acetaminophen and was discharged with amoxicillin for presumed pharyngitis.

Within 24 hours, however, she returned with rapidly progressive anterior neck and chest pain, worsening dyspnea, visible swelling extending to the sternal notch, tachycardia, tachypnea, and poor oral intake. At this point, given the rapid progression, neck and chest symptoms, and systemic illness, laboratory evaluation and cross-sectional imaging were obtained. Initial laboratory studies (Table 1) demonstrated leukopenia and lactic acidosis. The differential also described vacuolated neutrophils, supporting severe bacterial infection, although a consistent absolute neutrophil count was not available in the outside-hospital records.

**Table 1:** Initial laboratory results on presentation.

Laboratory parameter	Result	Reference range
White blood cell count (×10³/µL)	1.7	4.0-11.0
Serum lactate (outside-hospital evaluation) (mmol/L)	5.8	0.5-2.2
Peak serum lactate (early tertiary-center evaluation) (mmol/L)	7.4	0.5-2.2

**Figure 1:**
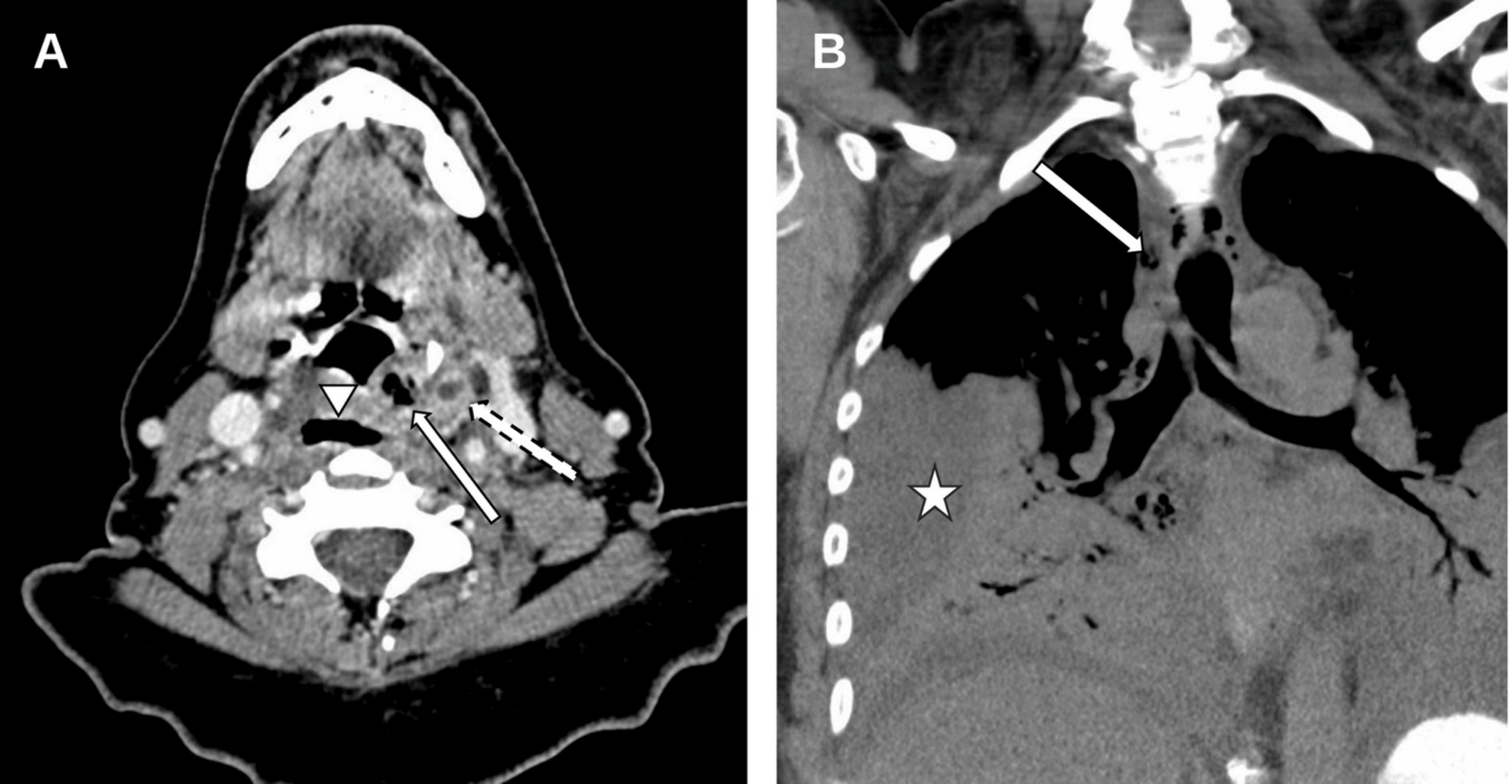
Initial CT imaging demonstrating descending necrotizing mediastinitis. (A) Contrast-enhanced axial CT neck shows extraluminal gas adjacent to the left hypopharynx/piriform sinus region (arrow) with retropharyngeal/prevertebral gas (triangle and dashed arrows). (B) Coronal CT chest shows pneumomediastinum (arrow) and pleural effusion (star).

Contrast-enhanced CT of the neck demonstrated gas throughout the prevertebral and parapharyngeal spaces extending into the superior mediastinum, with a linear focus of air and a small rim-enhancing fluid collection near the left hypopharynx (Figure 1A). Contrast-enhanced CT of the chest revealed pneumomediastinum with mediastinal fat stranding and bilateral pleural effusions (Figure 1B). No pulmonary embolism was identified. A contrast esophagram showed no leak from the thoracic esophagus. The working diagnosis shifted from uncomplicated pharyngitis to DNM, initially with concern for a cervical esophageal or hypopharyngeal perforation, and she was transferred urgently to a tertiary center.

On arrival, she was hypotensive and tachypneic with atrial fibrillation and elevated lactate. Bilateral chest tubes drained purulent fluid. She was intubated and started on broad-spectrum antimicrobials. She underwent emergent combined cervicothoracic drainage: upper endoscopy showed a normal esophagus without perforation; left cervicotomy revealed edematous tissues and thin, dishwater fluid tracking along the prevertebral space; and right thoracotomy drained similar pleural and mediastinal fluid. Closed-suction drains and chest tubes were placed, and DNM was diagnosed.

Pleural and mediastinal cultures grew *Streptococcus anginosus* and mixed oral flora, and blood cultures grew *Actinomyces odontolyticus*. Postoperatively, she developed profound septic shock with stress-induced cardiomyopathy, acute kidney injury requiring continuous renal replacement therapy, and symmetric peripheral ischemia while on vasopressors. She underwent repeat left neck exploration and debridement for recurrent cervical collections; the wound was left open for frequent antiseptic packing.

Because of prolonged postoperative ventilator dependence after septic shock and thoracotomy, the need for pulmonary toilet and airway protection, and inability to safely resume oral intake, tracheostomy and gastrostomy tube placement were performed during the second week of hospitalization.

On hospital day 17, repeat laryngoscopy and neck exploration identified a large left pharyngocutaneous fistula connecting the hypopharyngeal mucosa along the thyroid cartilage to the open cervical wound. Necrotic mucosa and soft tissue were debrided, the wound was opened widely, and antiseptic-impregnated gauze was placed into the cervical and mediastinal planes for bedside wound care.

After the necrotizing process resolved, the neck wound developed healthy granulation tissue, and there was no exposed major vessel. Because of critical illness and poor peripheral perfusion, regional or free flap reconstruction was considered high risk. The multidisciplinary team instead pursued a structured conservative pathway focused on local wound control, diversion of saliva, and suppression of salivary flow. The wound was packed multiple times daily with antiseptic-soaked gauze, with careful suctioning of saliva and exudate. She remained strictly nil per os with all nutrition delivered via gastrostomy. Systemic salivary suppression was initiated with glycopyrrolate and augmented with a scopolamine patch.

Serial laryngoscopies demonstrated gradual contraction of the mucosal defect. However, despite systemic anticholinergic therapy and meticulous wound care, salivary leakage remained substantial enough to require frequent repacking. Because of this persistent contamination, botulinum toxin A was injected into both parotid and both submandibular glands at approximately hospital day 40. Over the subsequent weeks, salivary output became easier to manage, and the external opening progressively contracted. The fistula closed by secondary intention over approximately 10 weeks without regional or free flap reconstruction.

Major milestones included tracheostomy and gastrostomy during the second week of hospitalization, fistula identification on hospital day 17, botulinum toxin injection at approximately hospital day 40, external closure by roughly 10 weeks, and endoscopic confirmation of healing at about four and a half months.

During her prolonged hospitalization, she ultimately required bilateral transmetatarsal amputations and multilevel amputations of all digits because of vasopressor-associated ischemia. At approximately three months after presentation, the cervical wound had closed externally, and there was no clinical evidence of persistent fistula. At about four and a half months after presentation, operative laryngoscopy confirmed a healed pharyngeal defect, and no further fistula-directed surgery was required.

## Discussion

This case illustrates two practical points: DNM can evolve rapidly after an initial presentation that appears benign, and large aerodigestive fistulas in a hostile neck may still close without flap reconstruction when conservative management is structured and prolonged.

DNM remains a high-mortality disease despite advances in imaging and critical care [2-7]. Outcomes depend on early recognition, prompt CT imaging of the neck and chest, broad-spectrum antibiotics, and aggressive drainage of both cervical and mediastinal disease [1-4,6,7]. In our patient, early symptoms were initially nonspecific and did not include the full constellation of classic deep-neck red flags, which likely contributed to the initial diagnosis of uncomplicated pharyngitis. Her subsequent rapid progression to neck swelling, chest pain, dyspnea, deep neck gas, pneumomediastinum, pleural involvement, and septic shock underscores how quickly DNM can evolve and why clinicians should maintain a low threshold for imaging when symptoms worsen over hours rather than days.

Pharyngocutaneous fistula is classically discussed after laryngectomy, where large or persistent defects, especially in irradiated or infected fields, are often reconstructed with regional or free tissue transfer [8,11]. However, multiple series and reviews suggest that optimized conservative care can close many fistulas, particularly when the wound bed is healthy, and major vessels are protected [8-12]. Conservative options described in the literature include enteral diversion, meticulous packing, salivary suppression, negative pressure wound therapy, and selected use of hyperbaric oxygen [8-12]. The available evidence is largely limited to small retrospective series and case reports, but these reports support the idea that nonoperative closure is feasible in appropriately selected patients [8-13]. Reports of pharyngocutaneous fistula in the context of necrotizing cervical infection or DNM remain limited [14-18].

Salivary control is a key lever for conservative fistula management. Systemic anticholinergics may be partially effective but can be limited by side effects and incomplete response. Botulinum toxin injections to the major salivary glands have been used to reduce salivary flow in refractory sialorrhea and in some post-laryngectomy pharyngocutaneous fistulas [9,15,16]. In our case, chemodenervation was added after weeks of persistent leakage despite maximal systemic therapy, and it coincided with improved local control and progressive contraction of the fistula. Although causality cannot be proven in a single case, salivary chemodenervation may be a useful adjunct when operative reconstruction is high risk and ongoing salivary contamination remains the main barrier to closure.

Negative pressure wound therapy was considered in the broader management framework but was not used in this case. Early in the course, the wound communicated with the hypopharynx and tracked into the mediastinum, required repeated direct inspection and serial debridement, and lay in proximity to major cervical vessels during the acute phase. For these reasons, open packing with frequent bedside assessment was favored until infection was controlled and a stable granulating wound bed had developed.

A conservative approach is not appropriate for every pharyngocutaneous fistula. In our patient, the decision was supported by resolution of necrotizing infection, a granulating wound bed, absence of exposed carotid or innominate artery, feasibility of enteral diversion, and serial laryngoscopies showing progressive improvement. By contrast, persistent high-output leakage, threatened vascular exposure, inability to control local contamination, or failure to show progressive healing on serial examinations would favor earlier regional or free flap reconstruction [8,11,17]. Our case, therefore, does not argue against flap reconstruction in general; rather, it suggests that carefully selected high-risk patients may still be candidates for a structured, time-limited trial of conservative care.

## Conclusions

DNM should remain in the differential diagnosis when a patient with a seemingly routine sore throat rapidly develops neck swelling, chest pain, dyspnea, or systemic toxicity. Early CT imaging of the neck and chest, and prompt multidisciplinary drainage are essential when mediastinal spread is suspected.

This single case does not diminish the role of regional or free flap reconstruction for many large pharyngocutaneous fistulas. However, it suggests that, in carefully selected high-risk patients, once infection is controlled, major vessels are protected, and the wound bed is healthy, meticulous wound care, strict salivary diversion, and salivary suppression may allow closure by secondary intention over time.
